# Human umbilical cord mesenchymal stem cells toxicity and allergy effects: In vivo assessment

**DOI:** 10.1371/journal.pone.0309429

**Published:** 2024-10-24

**Authors:** Wu Dong Cheng, Luo Bin, Zuo Xia Lin, Ding Yu, Ding Ke Xiang, Samantha Lo, Manickam Ravichandran, Tham Seng Kong

**Affiliations:** 1 School of Medicine, Wuhan University, Wuhan, Hubei, China; 2 ALPS Medical Centre, ALPS Global Holding, Kuala Lumpur, Malaysia; 3 Institute of Neurosciences, Guangzhou Medical University Hospital, Guangzhou, China; 4 Department of Health Management, Nanfang Hospital, Southern Medical University of China, Guangzhou, Guangdong, China; 5 School of Public Health, Southern Medical University of China, Guangzhou, Guangdong, China; 6 Celestialab Sdn Bhd, ALPS Global Holding, Kuala Lumpur, Malaysia; 7 MyGenome, ALPS Global Holding, Kuala Lumpur, Malaysia; 8 Faculty of Applied Sciences, Department of Biotechnology, AIMST University, Bedong, Kedah, Malaysia; Universita degli Studi di Perugia, ITALY

## Abstract

**Objective:**

Human umbilical cord mesenchymal stem cells (hUCMSCs) hold significant promise across various clinical applications. Therefore, regulatory requirements necessitate a thorough investigation of the hUCMSCs safety before clinical trials and potential allergic reactions after transplantation.

**Methods:**

Abnormal toxicity test employed mice and guinea pigs dosed daily at 0.5×10^6^ cells and 5×10^6^ cells, respectively for 7 days. Acute toxicity test employed low, medium, and high doses of hUCMSCs injected into mice once, followed by observations for 23 days. In systemic allergy test, guinea pigs received low and high dose of hUCMSCs, with sensitization and excitation stages at day 14 and 21, respectively.

**Results:**

The abnormal toxicity test of hUCMSC injections revealed no abnormal reactions over a seven-day observation period, indicating the safety of this administration route. In acute toxicity studies, the high-dose hUCMSCs group resulted in fatalities due to pulmonary embolism. Conversely, the low-dose group exhibited no toxic reactions or deaths. The maximum tolerated dose was determined to be >2×10^7^ cells/kg. Systemic active allergy test showed that high doses of hUCMSC intravenous injections did not induce allergic reactions.

**Conclusion:**

This research affirms hUCMSC injections meet safety standards for clinical cell therapy, emphasizing their safe and promising clinical utility.

## 1. Introduction

Human umbilical cord mesenchymal stem cells (hUCMSCs) are self-renewing and multipotent cells found in the umbilical cord tissue, capable of differentiating into osteoblasts, chondrocytes, and adipocytes, as well as secreting cytokines. These cells have shown promising therapeutic properties in various diseases due to their differentiation capabilities, immune regulation, paracrine effects, anti-inflammatory effects, etc [1]. Due to these unique characteristics, hUCMSCs can be utilised in the human body for an array of ailments, namely regenerative functions. However, ensuring the safety of hUCMSCs is crucial for advancing regenerative medicine. Comprehensive safety assessments, including toxicity and allergy effects, are vital for both preclinical and clinical applications [2]. Previous studies, such as Xie et al.’s evaluation system, demonstrated that qualified hUCMSCs had no severe adverse reactions during a 1-year follow-up [3]. Li et al. also found no toxicity on fertility or teratogenic effects in fetuses following intravenous injection of hUCMSCs [4]. These evaluations are essential for assessing safety and ensuring the responsible integration of hUCMSCs into clinical practice, bolstering regenerative medicine’s credibility [5]. The assessment of hUCMSC injections involves critical safety evaluations, including abnormal toxicity and acute toxicity tests, recognized worldwide as essential components for clinical injection safety assessments [6].

The abnormal toxicity test, conducted through intraperitoneal injection in mice and guinea pigs, aids in dosage selection, injection routes, and safety measures, adhering to internationally recognized good laboratory management practices (GLP) and new drug safety evaluation GLP specifications [7]. The evaluation emphasizes potential exogenous substance introduction and abnormal toxic reactions, linking injection quality to production technology proficiency and deviating from typical preclinical testing protocols [8]. In the Chinese Pharmacopoeia, abnormal toxicity tests are mandated in all biologics and vaccines for registration and lot release. This test also needs to be carried out using both mice and guinea pigs, per the guidelines set forth by the China Health Authority [9].

The acute toxicity test in KM mice observes toxic reactions and mortality from the maximum dose of hUCMSCs within a specific timeframe. It guides subsequent toxicity tests and determines initial doses for clinical trials, serving as a cornerstone in cell-based drug evaluation [10].

Evaluation of allergic reactions after hUCMSC transplantation employs the guinea pig whole-body active allergy test, assessing whether allogeneic stem cell injection may provoke allergic responses. The test involves intraperitoneal and intravenous injections, focusing on detecting abnormal immune or allergic responses and identifying antigens inciting allergic reactions [11]. In the Chinese Pharmacopoeia, the key abnormal toxicity tests focus on detecting unexpected toxic impurities in medicinal products for parenteral use and their active pharmaceutical ingredients [12].

For active systemic allergy testing, we employed the anaphylaxis test, which is mandated for special safety testing of national drug standard injections as outlined in China’s "Measures for the Administration of Drug Registration". This test involves sensitizing animals and intravenously injecting antigens to observe mast cell and basophil degranulation and active mediator release upon antibody binding, further emphasizing the importance of assessing allergic reactions in hUCMSCs’ biosafety evaluation [13].

The main objective of this study is to assess the biosafety and toxicity of hUCMSCs for clinical applications. It investigates biological characteristics post-amplification, examines potential allergic reactions post-transplantation, and determines the safety of hUCMSCs through abnormal toxicity and acute toxicity tests in animal models.

## 2. Materials and methods

All procedures in this study were conducted in accordance with the Committee on Animal Research and Ethics at The Second Affiliated Hospital of Guangzhou Medical University (Acceptance number: B2022-090) approved protocols. A total of 45 mice and 26 guinea pigs remained alive until the end of the experiments. After all experimental observations were completed within the stipulated timeframe, the animals were immediately anesthetized, followed by euthanization by exsanguination from the abdominal aorta. Histopathological examination was carried out immediately in case of visible changes or animal death. Animal health and behavior were monitored daily to ensure any signs of distress were promptly recorded as observational results and addressed. To minimize suffering and distress, analgesics and anesthetics were administered as necessary. All research staff received special training in animal care and handling to ensure proper welfare considerations throughout the study.

### 2.1 Experimental animals

Kunming mice (KM mice), belonging to the China Outbred group, are chosen for their widely recognized attributes in physiology, pharmacology, and toxicology. Their clear genetic background, high gene heterozygosity rate, disease resistance, adaptability, and other characteristics make them an internationally recognized and commonly used experimental animal strain [14]. KM mice are selected for conducting in vivo assessments of toxicity and allergy effects.

In contrast, guinea pigs, specifically the Hartley strain, are used in the abnormal toxicity test. Hartley guinea pigs, one of the four main breeds of British guinea pigs, are widely employed in experiments in China. They exhibit various fur colors and possess short and smooth fur, rapid growth, strong disease resistance, and stable gene frequencies. Due to their inability to synthesize vitamin C, they serve as an ideal model for studying scurvy and are commonly used in toxicity and allergy studies [15]. Table 1 demonstrates the number and types of animals used in this study.

**Table 1:** Number and types of animals used in this study.

Experiment	Type of Animal	Grouping		Total Number
		Experimental	Control	
**Abnormal toxicity test**	Kun Ming mice	5	5	10
	Hartley guinea pigs	2	2	4
**Acute toxicity test**	Kun Ming mice	3 groups, 10 mice each	10	40
**Active systemic allergy test**	Hartley guinea pigs	3 groups, 6 guinea pigs each	6	24

The number of animal subjects used and the hUCMSC quantity dosages assigned were based on the standards set in the Chinese Pharmacopoeia 2015 version [12].

### 2.2 Abnormal toxicity test

#### 2.2.1 Dosage and grouping method used in experiments

For the KM mice abnormal toxicity test, 10 specific pathogen-free (SPF)-class KM mice are randomly divided into two groups: the hUCMSCs experimental group and the normal saline control group, each containing 5 mice. In the hUCMSCs experimental group, each mouse receives a single intraperitoneal hUCMSC injection of 0.5mL at 1×10^6^ cells/mL, while in the normal saline control group, each mouse receives a single intraperitoneal injection of 0.5mL of sterile normal saline.

For the Hartley guinea pig abnormal toxicity test, 4 regular-grade Hartley guinea pigs are randomly divided into two groups: the hUCMSCs experimental group and the normal saline control group, each containing 2 guinea pigs. In the hUCMSCs experimental group, each guinea pig receives a single intraperitoneal injection of 5.0mL at 1×10^6^ cells/mL of hUCMSCs, while in the normal saline control group, each guinea pig receives a single intraperitoneal injection of 5.0mL of sterile normal saline.

The number of animal subjects used and the volume dosages given to both KM mice and Hartley guinea pigs were based on the standards set in the Chinese Pharmacopoeia 2015 version [12].

#### 2.2.2 Dosing regimen

The route of administration for the abnormal toxicity test in both mice and guinea pigs is intraperitoneal injection. The injection speed is slow, taking 0.5 minutes for each mouse and 3 minutes for each guinea pig to complete the injection. The administration frequency is once a day for both the hUCMSCs experimental group and the normal saline control group, and the dosing cycle is 1 day.

Daily observations and recordings were conducted continuously for 7 days. The observations focused on the fur color, eyes, breathing, secretions, excretions, autonomic and central nervous system behaviors of both guinea pigs and mice. Additionally, the number and time of animal deaths were recorded. Moreover, the body weight of the experimental animals was measured on the day of intraperitoneal administration and every day for the following 7 days.

### 2.3 Acute toxicity testing

#### 2.3.1 Experimental dosage design

The dosage design for hUCMSCs in this experiment is chiefly informed by the prevailing clinical dosages of hUCMSCs and the outcomes of preliminary experiments undertaken in the initial stages of this research. Specifically, for each clinical application, no fewer than 100×10^6^ hUCMSCs are transplanted, and this corresponds to a clinical dosage of 1.4×10^6^ cells/kg based on a 70 kg individual. Guided by pre-test results, the dosages were determined as follows: a low-dose group of 2×10^7^ cells/kg, a medium-dose group of 4×10^7^ cells/kg, and a high-dose group of 8×10^7^ cells/kg.

The choice of this test method not only complies with the national regulations of the Chinese health authority but also is more suitable for injectable drugs with low toxicity such as hUCMSCs. The maximum dosage method is based on the premise of a scientifically reasonable maximum dosage concentration and dosage, with the maximum allowed dosage administered once or multiple times within 24 hours, and observing the symptoms that appear in the experimental animals after administration [5].

#### 2.3.2 Experimental dosage and grouping

The organization of experimental animals in this study adheres to the foundational dosages outlined above. The specific approach to grouping experimental animals is as follows: The initial pool of 40 KM mice, meeting the specifications of SPF level, were distributed randomly into four groups based on their body weight. These groups are designated as the low-dose group (L), the medium-dose group (M), the high-dose group (H) of hUCMSCs, and a vehicle control group (C). The total of 40 KM mice in this experiment were apportioned evenly into four groups, each containing 10 experimental animals, with an equal distribution of females. Detailed information regarding the experimental dosages and grouping can be found in Table 2 below.

**Table 2:** Experimental dose and grouping information table.

Group	Test Material	Dosage (cells/kg)	Concentration (cells/mL)	Correspondence to Individuals in Clinical Multiples	Number of animals
**C**	Normal saline	0	0	0	10
**L**	hUCMSC	2×10^7^	1×10^6^	14.3	10
**M**	hUCMSC	4×10^7^	2×10^6^	28.6	10
**H**	hUCMSC	8×10^7^	4×10^6^	57.2	10

C: Control; L: Low-dose; M: Medium-dose; H: High-dose.

#### 2.3.3 Dosing regimen

Dosing route utilises injection into the tail vein of mice, slow injection, each mouse is injected in about 1 minute; dosing frequency: once in this test; dosing cycle: 1 day; Dosing volume: each experimental group was administered at a volume of 0.2 mL/10 g/bw per mouse.

### 2.4 Active systemic allergy testing

For this study, two cell concentrations were designed: a low-dose group and a high-dose group. The low-dose group of hUCMSCs has a cell density of 1×10^6^ cells/mL, while the high-dose group has a cell density of 2×10^6^ cells/mL, which is twice that of the low-dose group.

#### 2.4.1 Grouping of experimental animals

The 24 experimental guinea pigs are divided into four groups: low-dose hUCMSC group, high-dose hUCMSC group, normal saline negative control group, and ovalbumin positive control group. Each group contains six experimental guinea pigs, evenly divided between male and female individuals.

#### 2.4.2 Experimental dos

The hUCMSCs low-dose group has a sensitizing dose of 5×10^5^ cells/guinea pig and an excitation dose of 1×10^6^ cells/guinea pig. Calculated based on an approximate weight of 400g per guinea pig, the sensitizing dose is approximately 1.25×10^6^ cells/kg, and the excitation dose is approximately 2.5×10^6^ cells/kg. Similarly, the hUCMSCs high-dose group has a sensitizing dose of 1×10^6^ cells/guinea pig and an excitation dose of 2×10^6^ cells/guinea pig. Calculated using the same weight, the sensitizing dose is approximately 2.5×10^6^ cells/kg, and the excitation dose is approximately 5×10^6^ cells/kg. For the positive control group, ovalbumin is used, with a sensitizing dose of 5 mg/guinea pig and an excitation dose of 10 mg/guinea pig (Table 3).

**Table 3:** Experimental dosing regimen.

Group	Number of Guinea Pigs	Dosing Regimen	Sensitization Phase Dosage (injected on the 1^st^, 3^rd^, and 5^th^ day)	Excitation Phase Dosage (injected on the 14^th^ and 21^st^ day)
Low-dose hUCMSC	6	0.5 mL/guinea pig	5×10^5^ cells/guinea pig (1.25×10^6^ cells/kg)	1×10^6^ cells/guinea pig
High-dose hUCMSC	6	0.5 mL/guinea pig	1×10^6^ cells/guinea pig (2.5×10^6^ cells/kg)	2×10^6^ cells/guinea pig
Normal saline (negative control)	6	No hUCMSC injection	N/A	N/A
Ovalbumin (positive control)	6	No hUCMSC injection	5 mg/guinea pig	10 mg/guinea pig

#### 2.4.3 Experimental dosing regimen

The hUCMSCs systemic active allergy test in guinea pigs utilizes two administration routes for sensitization and excitation: intraperitoneal injection for sensitization and intravenous injection for excitation. The intraperitoneal injection route is commonly used for sensitization in allergy tests because it allows for the effective delivery of the allergen to the peritoneal cavity, where it can interact with immune cells and elicit an immune response [16]. Conversely, the intravenous injection route is chosen for the subsequent administration of the allergen to "excite" or challenge the immune system [17]. In the sensitization phase, albino guinea pigs are intraperitoneally injected with hUCMSCs on the 1st, 3rd, and 5th days. For the excitation phase, albino guinea pigs are intravenously injected with hUCMSCs on the 14th and 21st days after the last sensitization. The dosing frequency and cycle involve three days of sensitization in the sensitization stage and intravenous excitation injections on the 14th and 21st days. The dosing volume for intraperitoneal sensitization injection is 0.5 mL/guinea pig, while for intravenous excitation injection, it is increased to 1 mL/guinea pig.

### 2.5 Statistical analysis

The result data from this manuscript utilised repeated measures ANOVA (Analysis of Variance) for statistical analysis to produce graphical results.

## 3. Results

### 3.1 Abnormal toxicity test

Throughout the observation period of the abnormal toxicity test, which included the day of injection and the subsequent 7 days of observation, all the mice and guinea pigs in each test group showed normal signs and manifestations. There were no abnormalities observed in their coat color, eyes, breathing, secretions, excretions, autonomic and central nervous system behaviors. Additionally, none of the experimental animals died during the entire course of the experiment. This indicates that the intraperitoneal administration of hUCMSCs did not produce any abnormal toxicity or adverse effects on the experimental animals.

During the abnormal toxicity test, the experimental animals, both guinea pigs and mice, showed an increase in body weight from the day of hUCMSCs injection until the end of the observation period. Similarly, all five mice injected with hUCMSCs on day 1 (d1) showed an increase in body weight to day 7 (d7), and their body weight growth trend was similar to that of the normal saline control group. The observations indicate that the administration of hUCMSCs did not have a negative impact on the body weight of the experimental animals during the entire observation period (see Table 4).

**Table 4:** Guinea pig and mice weight gain results (g).

Group	Mean Weight Gain (g)	Standard Deviation of Weight Gain (g)
Guinea Pigs Injected with hUCMSCs	30.70	1.43
Control Group (Guinea Pigs)	20.34	8.69
Mice Injected with hUCMSCs	9.93	2.72
Control Group (Mice)	11.68	3.76

The weight gain of the experimental animals could be due to several factors. The initial weights of treatment groups may not be equal or nearly equal, leading to bias in treatment comparisons [18]. Additionally, variations in food intake and initial weights, even when controlled, can still result in small differences in weight gain [19]. It is also important to consider the animals may also adjust their foraging effort and alter energy reserves in response to changes in food availability [20].

#### 3.1.1 Anatomical observation results of experimental animals

During the entire observation period of the abnormal toxicity test, both guinea pigs and mice in each test group, from the day of hUCMSCs intraperitoneal injection until the end of the experiment, did not exhibit any abnormal changes or experience animal deaths. After the observation period, all animals were euthanized and underwent dissection, and a thorough visual examination was conducted on their tissues and organs (namely the heart, liver, spleen, lung, kidney, and brain). No toxic side effects or abnormal changes were observed in any of the tissues and organs of both the guinea pigs and mice. This means that the animals in the experimental group, which received hUCMSCs injection, showed similar results to those in the control group that received normal saline injection, and no toxic side effects or abnormal changes were detected in any of the tissues and organs examined.

### 3.2 Acute toxicity test

#### 3.2.1 Observation results of the death of experimental animals and general symptoms

Human umbilical cord mesenchymal stem cells were injected into the experimental animals through the tail vein of the experimental animals respectively. Observation of general symptoms, signs and animal death on the day: 4 animals in the hUCMSCs high-dose group animals, one experimental animal in the hUCMSCs medium-dose group died within half an hour after hUCMSCs injection; moreover, the rest of the hUCMSCs high-dose group and the medium-dose group also showed varying degrees of shortness of breath with symptoms such as decreased activity and sluggish response. However, these symptoms all returned to normal within 1 hour. The experimental animals in the medium dose group died on the second day after intravenous injection, and the cause of death may be related to animal injury from the operator. In addition, all experimental animals in the hUCMSCs low-dose group did not show any abnormal reaction or death after tail vein injection and during the observation period of 23 days after injection. No animal death or other abnormalities occurred in the remaining experimental animals during the observation period of 23 days after injection.

#### 3.2.2 The body weight change results of the experimental animals

The body weight of the high-dose group on the first and second day after the treatment were both significantly different (P<0.01, P<0.05 respectively) from the vehicle control group. The body weights of the other groups were lower than those of the vehicle control group; the body weights of other groups were not significantly different from those of the vehicle control group at each stage (P>0.05). See Table 5 for details.

**Table 5:** Body weight change results of experimental animals (KM mice) ($\bar{x}\pm s$, n = 10, g).

Time (d)	Solvent control group	Low dose group	Medium dose group	High dose group
0	19.71±1.44	19.48±0.79	19.39±0.98	19.24±0.73
1	20.67±1.46	20.24±0.93	19.23±1.38 a	18.30±1.32** c
2	22.42±1.86	22.18±1.08	20.55±2.00 a	20.30±1.41* c
3	23.88±2.29	23.88±1.99	21.86±2.31 b	21.47±1.42 c
7	29.68±4.61	28.60±3.05	27.52±3.57 b	28.41±2.30 c
14	36.15±4.20	35.07±4.03	32.02±4.83 b	34.58±4.32 c
23	38.44±4.04	35.91±2.14	35.45±4.77 b	37.42±5.81 c

Note: Compared with vehicle control group

*P <0.05

**P <0.01; a: n = 9, b: n = 8, c: n = 6.

#### 3.2.3 Histopathological examination results of experimental animals

After conducting pathological anatomical examination of the main organs of the dead animals during the experimental observation period, the tissues and organs of the animals in each experimental group were carefully observed with the naked eye, and no gross abnormal changes were found. Several vital organs (heart, liver, spleen, lung, kidney, brain) of dead animals were examined histopathologically. The results indicated that, during the experimental observation period, microthrombosis was detected in the small pulmonary vessels and capillaries of the experimental animals. Additionally, on the second day after intravenous injection, small pulmonary vessels and capillaries exhibiting microthrombosis were observed in the deceased animals from the medium dose group of hUCMSCs. Microthrombosis is accompanied by slight degeneration of liver cells; the pathological anatomy of the experimental animals after the end of the observation period was carefully observed with the naked eye, no abnormal changes were found in the animal organs of each experimental tissue, and then the animal organs of each experimental tissue were examined.

For detailed histopathological examination report, see S1 Appendix.

### 3.3 Active systemic allergy test

#### 3.3.1 Body mass (body weight) measurement results of different groups of test animals at different stages

**Fig 1:**
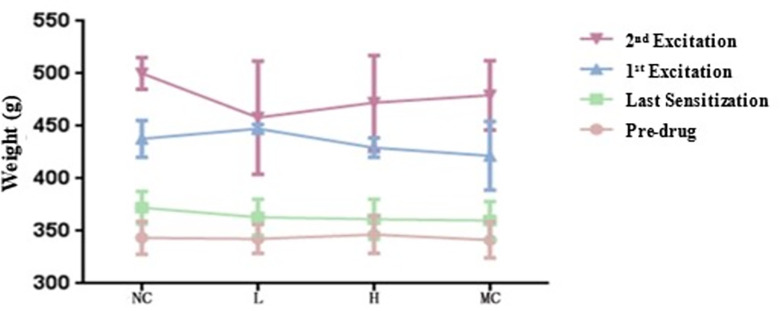
Body weight measurements of different groups of test animals at the same stage ($\bar{x}\pm s$) curve.

**Fig 2:**
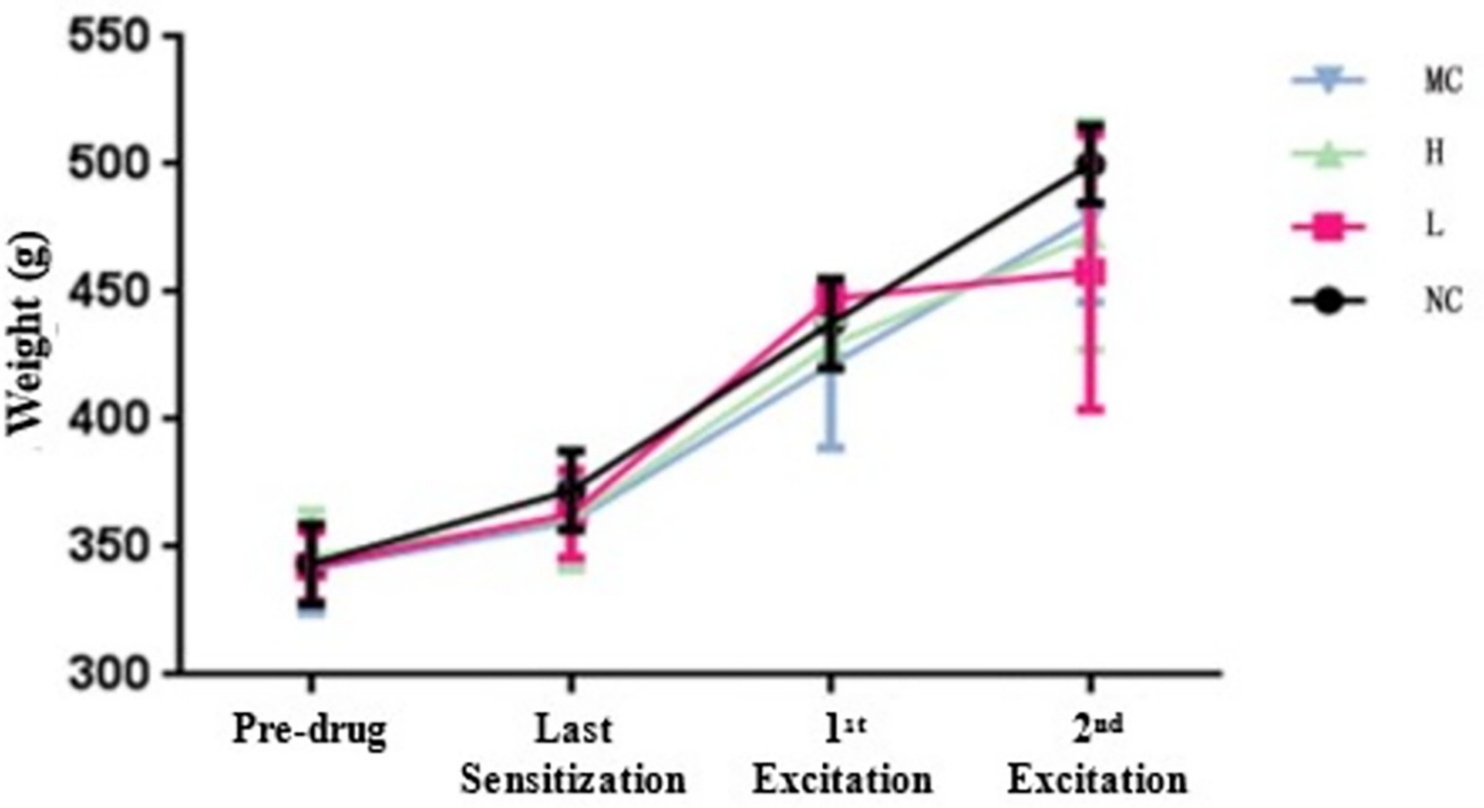
Body weight measurements of the same group of test animals at different stages ($\bar{x}\pm s$) curve.

The body weights of various test animal groups were measured at different stages. Before administering the first intraperitoneal injection, the body weights of all the tested guinea pigs in the experimental group receiving low-dose hUCMSCs, negative control saline, and positive control ovalbumin were recorded on the day of the last sensitization, the first challenge, and the second challenge. The results showed no significant difference in body weight among the measured values (refer to Table 6 and Fig 1) with a P-value less than 0.05. This suggests that the injections of different preparations did not notably affect the test animals’ body weight under the experimental conditions. Additionally, within the same group and across different stages (pre-drug, last sensitization, first challenge, second challenge), the body weight of the test animals increased as the duration of experimental feeding time extended. This pattern aligns with the typical growth characteristics and laws observed in experimental animal growth (Fig 2).

**Table 6:** Body mass (body weight) measurement results of different groups of test animals at different stages ($\bar{x}\pm s$).

Stage	Number of animals	Group			
		NC	L	H	MC
Pre-drug	6	343.58±15.48	342.62±13.81	346.73±17.81	341.66±17.20
Last Sensitization	6	372.40±15.49	363.30±17.21	361.40±18.93	360.07±18.16
1^st^ Excitation	3	438.02±17.46	447.59±4.26	429.56±9.32	421.80±32.63
2^nd^ Excitation	3	500.32±15.25	458.15±53.84	472.40±44.90	479.39±32.97

*NC: Negative control group; L: Low-dose group; H: High-dose group; MC: Positive control group.

#### 3.3.2 Observations and outcomes of symptoms leading to death during the sensitization stage of animals in different groups

Starting from the sensitization phase of guinea pigs, we meticulously monitored and recorded any allergic symptoms in the guinea pigs every day. This included a total of twenty-four guinea pigs at the onset of the experiment, which received intraperitoneal injections on days 1, 3, and 5. The injections consisted of low-dose (5x10^5^ cells per injection) and high-dose (1x10^6^ cells per injection) hUCMSCs, saline injections (0.9% NaCl), and ovalbumin injections (5mg per injection) in 0.5mL volumes. We closely observed the mental state, spontaneous activity, reaction strength, eye secretions, eating, drinking, defecation, and excretion of the guinea pigs in the low-dose hUCMSC group, high-dose hUCMSC group, positive control group, and negative control group during and after sensitization. Fortunately, no abnormalities, allergic reactions, or animal deaths were observed in any of these groups.

#### 3.3.3 Symptoms and outcomes of death in the challenge stage for different groups of animals

On the 14^th^ day after intraperitoneal injection of sensitization on the 5^th^ day, 3 guinea pigs in each group were injected intravenously with hUCMSCs at a low cell dose (1×10^6^ cells/animal) and a high cell dose (2×10^6^ cells/animal). 1.0 mL each of physiological saline (0.9% NaCl) and ovalbumin injection (sensitizing dose: 10 mg/pig); at the same time, all remaining guinea pigs in each group were intraperitoneally injected on the 5^th^ day mentioned above on the 21^st^ day after sensitization. Use the same specifications and types of experimental preparations and follow the same experimental methods for stimulation. After careful and careful observation of the allergic reactions of the guinea pigs in each group, the animals in the hUCMSCs high- and low-dose groups and the negative control group were stimulated. All guinea pigs died within 30 minutes, and no allergic symptoms were observed. The active allergy test was negative and the reaction was normal; however, after the stimulation of the 6 guinea pigs in the positive control group, all of the test animals experienced tremors, 4/6 guinea pigs had difficulty urinating and breathing, and 2/6 guinea pigs developed nose scratching, defecation, unsteady gait, and convulsions. Afterwards, all guinea pigs died within 4 minutes. The positive reaction rate was 100%.

## 4. Discussion

The abnormal toxicity test assesses injection medicine in experimental animals to check compliance with regulations and identify highly toxic impurities [21]. A failed test deems the injection unsuitable for clinical use, emphasizing production processes over inherent toxicity. This testing, distinct from acute preclinical drug testing, demands high sensitivity, precision, and reproducibility [22]. Abnormal toxicity tests on experimental animals are crucial for the safety of new clinical drug injections, with the Chinese Pharmacopoeia providing clear regulations [9]. To ensure the safety of hUCMSCs injection, this report follows the Chinese Pharmacopoeia 2015 guidelines, using an experimental dose over 10 times the clinical adult dose in mice and guinea pigs observed over a 7-day cycle, aligning with national health department requirements [23].

Throughout the observation period, there were no abnormal changes or animal deaths in any of the test groups, whether mice or guinea pigs. All experimental animals in the hUCMSCs injection group as well as the control group showed no abnormal signs in coat color, eyes, breathing, secretions, excretions, autonomic and central nervous system behaviors. No toxic side effects or abnormal changes were observed in various tissues and organs during the dissection and examination of the experimental animals after the test. These results indicate that the hUCMSCs injection preparation demonstrated no abnormal toxicity in the tested animals and met the safety requirements for clinical application. The rigorous abnormal toxicity testing performed in this study contributes to ensuring the biological safety of clinical medications and treatments.

In acute toxicity test of hUCMSCs, all experimental animals in the hUCMSCs medium-dose group and high-dose group showed reduced activity, sluggish response and other abnormal conditions, and even the death of individual experimental animals. The cause of death of these experimental animals were analyzed and found microthrombosis in small blood vessels and capillaries in the lungs [24]. All experimental animals in the low-dose hUCMSCs injection group did not have any abnormal reaction or death after tail vein injection and during the 23-day observation period of injection. No animal death or other abnormalities occurred in the remaining experimental animals during the observation period of 23 days after injection. This shows that in the future clinical application of hUCMSC injection, a clinical application level must be used, and the cell density and total number of cells infused with hUCMSCs must be strictly controlled, as well as a steady and slow injection speed should be maintained. The application of hUCMSCs is considered safe when a scientifically reasonable and safe cell concentration or density is ensured [3].

In other literature, hUCMSCs have been studied for their potential toxic effects in various animal models. In a similar study on the toxicity of hUCMSCs in mice and rats, acute toxicity was assessed in mice through two caudal intravenous injections at a maximum tolerated dose of 1.5×10^7^ cells/kg, with no observed acute toxicity. For long-term toxicity in rats, groups were treated with hUCMSCs at different doses comprising of low-dose (3.0×10^5^ cells/kg), mid-dose (1.5×10^6^ cells/kg), and high-dose (7.5×10^6^ cells/kg) via intravenous injection every 3 days for 90 days. There were no significant differences in body weight, hematological and blood biochemical parameters, or histopathologic changes in vital organs between hUCMSC-treated and control groups [25]. In comparison, the maximum tolerated dose as well as the different doses of hUCMSC used were much greater in the present study than the literature research data, indicating greater tolerance of the experimental animals towards hUCMSC concentrations.

In Sprague-Dawley rats, intravenous injection of hUCMSCs did not show any toxicity on fertility or teratogenic effects on fetuses [4]. However, higher doses of hUCMSCs caused mild decrease in body weight gain in male rats and symptoms like listlessness, tachypnea, and hematuria in pregnant female rats [26]. In mice, hUCMSCs demonstrated hepatoprotective effects without significant toxicity or tumorigenicity [27]. In guinea pigs, hUCMSCs were found to migrate to the nasal mucosa lamina propria and improve mucus clearance time and mucosal edema within a short time period. However, they did not differentiate into nasal epithelial cells or improve nasal epithelial regeneration in the long term [4].

In active systemic allergy testing, the experiment conclusively establishes that hUCMSCs, when applied in both low-dose and high-dose groups, do not elicit active systemic allergic reactions or lead to guinea pig fatalities due to systemic allergy. Additionally, the guinea pig body weight results indicated that all hUCMSC test groups and control groups exhibited no significant weight changes within the same experimental stage. This substantiates that the administration of hUCMSCs, under the provided test conditions, not only yielded a negative (normal) guinea pig systemic active allergic reaction but also exhibited stability in body weight across experimental groups and stages.

In contrast, the positive control group, stimulated with ovalbumin via intravenous injection, produced positive systemic allergy results. Within a mere 4 minutes of stimulation, all six guinea pigs in this group exhibited allergy symptoms, resulting in a 100% positive sensitization rate. These symptoms encompassed trembling, nose scratching, shortness of breath, urination, defecation, dyspnea, unsteady gait, and convulsions, culminating in the mortality of all guinea pigs within the same 4-minute timeframe [28]. Ovalbumin, recommended as a positive substance for inducing systemic active allergic reactions in guinea pigs, was administered at a dose of 2 mg per guinea pig [29]. This dose consistently induced comprehensive positive reaction symptoms on both the 14^th^ and 21^st^ days post-sensitization.

While no published studies have discussed on hUCMSC system allergy effects on guinea pigs, safety studies of transplantation of exosomes derived from hUCMSC found no allergic reactions in rabbits, guinea pigs, and rats [30].

## 5. Conclusion

In summary, the abnormal toxicity test and acute toxicity test conducted on hUCMSCs affirm their safety and suitability for clinical use. The abnormal toxicity test, administered intraperitoneally to mice and guinea pigs, revealed no abnormal reactions, toxic side effects, or deaths, indicating compliance with international and domestic regulatory standards. The acute toxicity test, involving high-dose injections into the tail vein of mice, demonstrated a maximal tolerated dose of 2×10^7^ cells/kg without inducing toxicity or fatalities. Guinea pig sensitization tests further confirmed the absence of active systemic anaphylactic reactions or weight fluctuations, supporting the conclusion that hUCMSCs meet the requirements for clinical human injections. These comprehensive assessments underscore the safety of hUCMSCs, positioning them as promising candidates for clinical applications.

## Supporting information

S1 Appendix Pathological examination report of human umbilical cord mesenchymal stem cells single intravenous injection toxicity experiment. (DOCX)
