# Supplementary material for: Human umbilical cord mesenchymal stem cells toxicity and allergy effects: In vivo assessment
Source: PLoS One. 2024 Oct 24;19(10):e0309429. doi: 10.1371/journal.pone.0309429 (PMC11500854; doi:10.1371/journal.pone.0309429)
Supplement: S1 Appendix — (DOCX) [file pone.0309429.s001.docx]

**S2 Appendix**

**Pathological examination report of human umbilical cord mesenchymal stem cells single intravenous injection toxicity experiment**

40 SPF grade KM mice were divided into vehicle control group, human umbilical cord mesenchymal stem cell low, medium and high dose groups (2x10^7^, 4x10^7^, 8x10^7^ cells/kg), 10 animals in each group, 5 male and 5 female. Intravenous single injection of the test product, 5 animals died on the same day after the administration, 1 male animal at the medium dose, 1 female animal and 3 male animals at the high dose; on the second day after the administration, 1 male animal at the medium dose died. Gross necropsy of all dead animals showed no obvious abnormal changes. The remaining 34 animals were continuously observed for 23 days after administration, and pathological anatomy was performed after bloodletting under anesthesia. All surviving animals in the administration group had no obvious abnormal changes with the naked eye. In order to find out the cause of death of the animals, the heart, liver, spleen, lung, kidney and brain tissues of the 6 dead animals were taken for histopathological examination. The results showed that microthrombosis was found in the small blood vessels and capillaries of the lungs of the 6 animals. In addition, the drug A male animal with a moderate dose died on the second day afterward, with scattered fresh hemorrhages in the liver and slight degeneration of focal hepatocytes. The rest of the organs showed no obvious abnormalities.

Key words: Human umbilical cord mesenchymal stem cells; KM mice; acute toxicity experiment; histopathological examination

**1. Purpose**

Observe the single intravenous injection of human umbilical cord mesenchymal stem cells into KM mice, and observe whether there are any abnormalities in various organs and tissues by gross necropsy, and preliminarily judge its possible toxic target organs.

**2. Materials and methods**

**2.1 Experimental animals and methods**

40 SPF grade KM mice were divided into vehicle control group, human umbilical cord mesenchymal stem cell low, medium and high dose groups (2x10^7^, 4x10^7^, 8x10^7^ cells/kg), 10 animals in each group, 5 male and 5 female, given hUCMSCs by intravenous single injection. All dead and living animals (After intraperitoneal anesthesia with 0.4% pentobarbital sodium, the abdominal aorta was exsanguinated) for pathological examination.

**2.2 Histopathological examination**

All animals were systematically dissected and pathological changes were recorded whether there were any abnormal changes in the organs and tissues were observed with the naked eye. If there were visible changes in the tissues and organs, the histopathological examination was carried out. Important organs including heart, liver, spleen, lung, kidney and brain tissue were taken from 6 dead animals for histopathological examination to determine the cause of death.

**3. General observation results of organ anatomy**

**3.1 Examination of dead animals**

During the single high-dose intravenous drug toxicity test of hUCMSCs, a total of 6 experimental animals died, mainly in KM mice in the high-dose hUCMSCs group and some medium-dose groups. Among them, 5 animals died on the day of hUCMSCs injection, including 1 animal (♂23) in the medium dose group and 4 animals in the high dose group (♀6, ♂22, ♂25, ♂37). Later, on the second day after hUCMSCs injection, another experimental animal in the medium dose group died (♂38). However, all the dead animals were dissected, and no obvious abnormalities visible to the naked eye were found in the gross tissue and organ observation.

**3.2 Examination of euthanized animals**

After the experimental observation period ended, all the remaining 34 experimental animals were euthanized, and then anatomical examination was performed, and no abnormal changes visible to the naked eye were observed in the general inspection of various tissues and organs.

**4. Histopathological examination**

 On this basis, histopathological examination showed that in the 5 animals that died on the day of intravenous injection of hUCMSCs, microthrombosis could be seen in the small blood vessels and capillaries of the lungs, but the heart, liver, spleen, kidney, brain, etc. There were no obvious abnormalities in the vital organs; in addition, in another experimental animal that died on the second day after hUCMSCs intravenous administration, microthrombosis could be seen except for small blood vessels in the lungs. In liver tissue, a single lobe of liver was scattered with fresh hemorrhage and slight degeneration of focal liver cells, while the rest of the heart, spleen, kidney, and brain showed no obvious abnormalities.

**5. Discussion and conclusion**

During the single high-dose intravenous drug toxicity test of hUCMSCs, hUCMSCs low dose group, no animal died, and no obvious abnormalities were found in gross anatomy; 2 animals in the hUCMSCs medium dose group died, 4 animals in the high dose group died, and no obvious abnormal changes were found in the gross necropsy of the dead animals. Histopathological examination results showed that microthrombosis was found in small blood vessels in the lungs of the 6 dead animals, and 1 animal had focal fresh hemorrhage in the liver and slight degeneration of liver cells, with the lesion confined to only a single liver lobe. This should not be related to the injection of the test product and may have been caused by the trauma from grasping the animal too tightly. The surviving animals in the medium dose group and high dose group showed no obvious abnormal changes in gross autopsy.

**6. Experimental pictures of histopathological examination**
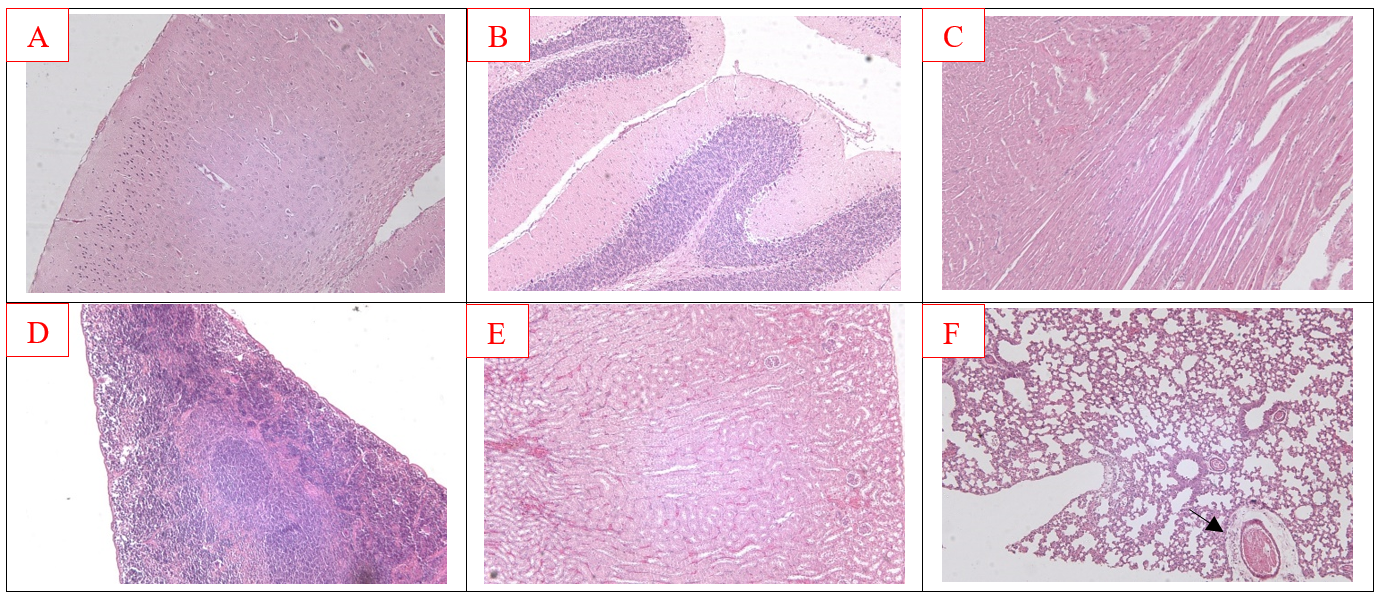

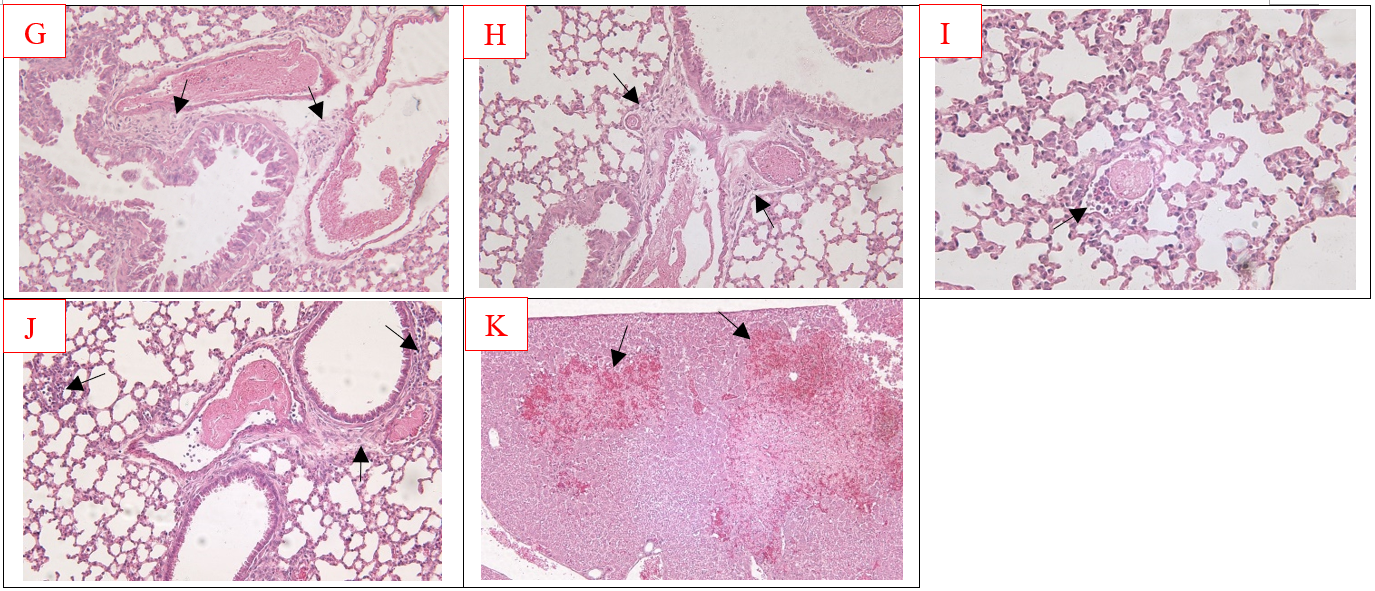
Figures A, B, C, D, E, F depicted the histological staining results of the medium dose groups captured at 10X objective lens for the brain, cerebellum, myocardium, spleen, kidney, and pulmonary blood vessels, respectively. No abnormalities were observed from figures A to E, however figure F displayed microthrombosis. Figures G and H depicted histological staining results of the high dose group at 20X objective lens for pulmonary blood vessels with visible microthrombosis. Figures I and J demonstrated microthrombosis present in pulmonary blood vessels and lung blood vessels, respectively, observed at 40X objective lens. Figure K showed the scattered fresh haemorrhage in the liver and slight degeneration of liver cells, seen at 10x objective lens.
